# Role of *Phaseolus vulgaris* L. in the Prevention of Cardiovascular Diseases—Cardioprotective Potential of Bioactive Compounds

**DOI:** 10.3390/plants11020186

**Published:** 2022-01-11

**Authors:** Lyanne Rodríguez, Diego Mendez, Hector Montecino, Basilio Carrasco, Barbara Arevalo, Iván Palomo, Eduardo Fuentes

**Affiliations:** 1 Thrombosis Research Center, Department of Clinical Biochemistry and Immunohematology, Faculty of Health Sciences, Medical Technology School, Universidad de Talca, Talca 3460000, Chile; lyrodriguez@utalca.cl (L.R.); diego.mendez@utalca.cl (D.M.); hector.montecino@utalca.cl (H.M.); 2 Centro de Estudios en Alimentos Procesados, Talca 3460000, Chile; bcarrasco@ceap.cl (B.C.); barevalo@ceap.cl (B.A.)

**Keywords:** cardiovascular diseases, *Phaseolus vulgaris* L., beans, bioactive compounds, cardioprotective

## Abstract

In terms of safe and healthy food, beans play a relevant role. This crop belongs to the species of *Phaseolus* *vulgaris* L., being the most consumed legume worldwide, both for poor and developed countries, the latter seek to direct their diet to healthy feeding, mainly low in fat. *Phaseolus vulgaris* L. stands out in this area—an important source of protein, vitamins, essential minerals, soluble fiber, starch, phytochemicals, and low in fat from foods. This species has been attributed many beneficial properties for health; it has effects on the circulatory system, immune system, digestive system, among others. It has been suggested that *Phaseolus vulgaris* L. has a relevant role in the prevention of cardiovascular events, the main cause of mortality and morbidity worldwide. Conversely, the decrease in the consumption of this legume has been related to an increase in the prevalence of cardiovascular diseases. This review will allow us to relate the nutritional level of this species with cardiovascular events, based on the correlation of the main bioactive compounds and their role as cardiovascular protectors, in addition to revealing the main mechanisms that explain the cardioprotective effects regulated by the bioactive components.

## 1. Introduction

Cardiovascular disease (CVD) is the leading cause of death in modern societies [1] and has a substantial economic impact [2]. CVD claimed more than 10 million lives in the last 18 years [3]. Conversely, many people suffer disability after suffering cardiovascular events [4]. Ninety percent of CVD deaths are attributed to conventional cardiovascular risk factors (CVRF) [5,6], which increase the probability of suffering from CVD [7]. There are non-modifiable CVRFs such as age and genetic predisposition [8,9]. Conversely, there are modifiable CVRFs: smoking, dyslipidemia, arterial hypertension, diabetes, physical inactivity, and overweight/obesity [5,6].

Platelets are considered the main pathological risk factors for CVD, such as coronary artery disease and atherosclerosis [1]. They are known for their fundamental contributions to thrombosis and hemostasis [10,11]. Platelet activation plays a fundamental role in the development of arterial thrombosis, for which the control of platelet function is essential for the prevention of thrombotic events [12].

Diet and lifestyle are modifiable risk factors that can have a significant impact on the probability that an individual develops different diseases [13,14,15].

Nutritional status is an important factor in preparing the immune system to fight infection or prevent non-communicable diseases [13,16]. Diet can have beneficial effects on several CVD risk factors [2], due to its cardioprotective and antiplatelet effects in the primary and secondary prevention of CVD [1]. Certain components of the diet with antiplatelet activity can reduce blood platelet activation and have an important influence on the treatment of cardiovascular events [2]. It has been described that there is an inverse association between antioxidants from the diet and the development of thrombosis and other coronary events [12,17,18].

Many foods are considered functional because they provide nutrients and energy to support daily life [19]. Grain legumes contain high amounts of protein, minerals, and vitamins and play an important role in both agricultural systems and the human diet, mainly in developing countries [20,21]. Out of 13,000 species, only between 10 to 12 play a relevant role today, either for application in the food industry or other commercial purposes [22,23]. Among them, we can mention common beans (*Phaseolus vulgaris*), chickpeas (*Cicer arientinum* L.), lentils (*Lens esculenta*), peas (*Pisum sativum*), and broad beans (*Vicia faba*) [21].

Grain legumes are a considerable source of nutrients and have also been referred to as the meat of the poor [24], because of their importance for consumption in third world, countries where malnutrition is a relevant nutritional problem [25,26]. In general, beans are recognized as a good source of protein, 2–3 times greater than that of cereal grains [26,27]. In ancient times, protein of animal origin was consumed mainly. Recently, there is a trend toward a Mediterranean diet, which includes foods of plant origin rich in nutrients that help reduce cholesterol levels in the population, among other health benefits [1].

Thus far, there are not many reports of the natural bioactive components with cardioprotective activity present in *P. vulgaris*. Certainly, elaborating on the subject could be a promising target in the prevention of CVD. The research aims to improve the lifestyle of the population by showing the nutritional value of beans and how this composition can be related to cardiovascular protection, thus promoting the consumption of this legume with properties that favor the maintenance and functioning of the body.

A literature review of research articles and bibliographic reviews was carried out in bibliographic databases: Pubmed, ScienceDirect, and GoogleScholar. Articles published between 2000 and 2021 were mainly selected, using terms: CVD; *Phaseolus vulgaris* L.; beans; bioactive compounds; cardioprotective; nutritional composition. Key articles were selected in the reference section of the original research to broaden the search. The inclusion criteria were all articles in English; some in Portuguese were also reviewed.

## 2. *Phaseolus vulgaris* L.

The common bean (*Phaseolus vulgaris* L.) is known as a “new world crop”; it originated 7000 years ago in two different parts of the North and South American continents [19,28,29] 

It belongs to the genus *Phaseolus*, family Leguminosae, subfamily *Papilionoideae*, tribe *Phaseoleae*, subtribe *Phaseolinae*; it is the legume species with the highest distribution and consumption of genus *Phaseolus*, which comprises about 70 species [29]. It has five cultivars domesticated in the pre-Columbian era, domesticated species: common bean (*Phaseolus vulgaris* L.), lima bean (*P. lunatus* L.), red bean (*Phaseolus coccineus* L.), tepary bean (*P. acutifolius Gray A*.), and beans (*P. polyanthus Greenman*), which present different adaptations and reproductive systems: mesic and temperate, predominantly self-pollinated [29,30].

*Phaseolus vulgaris* L. represents more than 90% of the crop grown in the world [29,31]. It is considered a non-centric crop with at least two domestication centers [32] and wide geographic distribution of its wild relatives in Central and South America. This crop was domesticated from wild *Phaseolus vulgaris* L., an indeterminate viniferous plant, distributed from Mexico to Argentina, mainly in mid-altitude neotropical and subtropical regions [33]. This species is an important food for rural and urban populations, mainly in Latin America and East Africa, although its demand has currently increased in developed countries, where populations are worrying about maintaining healthier diets [32]. *Phaseolus vulgaris* L., compared to other food crops, shows great diversity in terms of growth habits, seed characteristics (size, shape, and color), maturation times, and adaptation [34].

The common bean is the most important legume in the world for both human consumption and animal feed [30]. Its consumption is aimed especially at low-income people [25]. In areas such as Mexico, Central, and South America, and African countries, there is a high consumption and are considered as staple foods, considering a per capita intake of up to 40 kg per year [26,35,36]. The leading countries in the production of these legumes are Latin America and sub-Saharan Africa, where three-quarters of this crop is grown, with a production of around 12 million metric tons per year [20,37]. The forms of consumption are varied; consumers of beans from different countries and regions, even within the same country, show different predilections according to the size, shape, and color of the seed, as well as cooking time, the appearance of the broth and shape of storage [38,39].

*Phaseolus vulgaris* L. is consumed mainly by its dry grains (ripe), peel beans (seeds in physiological maturity), and green pods [30,33]. Beans are not only used as a dry grain, green beans are consumed as vegetables [29]. The seeds can be used in multiple ways, such as whole unprocessed seeds, as part of mixes, canned goods, or as a substitute for gluten-free wheat flour [19]. The United Nations for Food and Agriculture (FAO) in 2016 reported world production of a dry grain of 26.8 million tons, while the production as vegetable or green bean was 23.5 million tons. The most important classes of dried beans include green beans [26], red kidney beans [40], black beans [41], beans Mexican [42], pinto beans, tirage beans [43], great northern beans, navy beans, and pink beans [26,36].

Subtypes of Bean in Chile

Despite the nutritional value of beans and their consumption in Chile, there are few studies of these crops, which date back to before 2000. Food entities recommend this product as a component of a safe diet. Several cultivars are spread and consumed throughout Chile, achieving a high impact on the national diet [21]. The consumption of beans in Chile is under 1.5 kg per capita/year [44], in comparison with the 10 to 17 kg per capita/year consumed in Central American countries, and more than 50 kg per capita in some African countries [45].

The common bean germplasm collected in Chile has been classified as races Chile, Nueva Granada, Peru, Durango, and Mesoamerica, with the only exception to the race Jalisco. The Chilean strain has distinguished itself as an important source of genetic diversity [46]. This situation could be associated with commercial reasons and mainly due to the excellent adaptation of bean species to Chilean agro-climatic conditions. The current bean collection in Chile consists of 1110 accessions [38].

In most Chilean markets, the most consumed dried beans are Tortola and Coscorrón [47]. In some specific rural areas other crops, Manteca, Sapito, and Cuyano, are also consumed regularly [48]. There are other types consumed on a smaller scale, such as “bayos” and “sulfur”, all of a single seed color, but it is also common to find grains of two or more colors, such as “strawberries”, “araucano” and “sapito”, among others [49]. Dry grain preferences in Chile are directed mainly to the texture of the cooked grain.

The Chilean race has been characterized as a sub-center of genetic diversity. A distribution analysis comprised 1239 accessions that evaluated the genetic diversity present in 11 morphological characters. Great growth variability was evidenced (leaf, flower color from white to purple, presence of all types of bracteoles, diversity of shape, size, and color of pods with dorsal or central apex). The seed showed variations in size (small to large), shape (round to elongated), and great variation in the primary color or their combination. These results were useful for the genetic improvement of “tórtola” and “coscorrón” types [49]. A recent study suggests that the Chile race would be the oldest reservoir of genetic diversity in the Andean pool, making this germplasm a relevant genetic resource [50].

There is little information on the content of minerals, flavonoids, phenolic acids, total phenols, tannins, cooking quality, and antioxidant capacity of common beans of the Chile breed [38]. A study carried out by Paredes et al., 2009, evaluated the macro and micronutrient variability of a representative sample of beans from a Chilean breed collection, comparing them with representatives of other breeds. The results showed the existence of a wide variability for some macro and micronutrients, such as N, Fe, and Zn. The protein content ranged from 183.5 to 259.7 mg kg^−1^, Fe from 68.9 to 152.4 mg kg^−1^, and Zn from 27.9 to 40.7 mg kg^−1^. The bean genotypes of the Chile breed showed a good level of protein, Fe, and Zn; they did not show significant differences with the genotypes of other breeds [38]. This study allowed the selection of outstanding crops within the Chilean breeds studied, also allowing to improve current crops.

The National Institute of Agricultural Research (INIA) some years ago evaluated the proximal chemical composition and the biological quality of the protein of five new cultivars in comparison with two traditional cultivars of *Phaseolus vulgaris* L. The beans provided a large fraction of proteins and other nutrients. Dried beans also stood out for the nutritional quality of their protein, carbohydrates, minerals, and dietary fiber [21].

## 3. Relationship between Nutritional Composition and Health Benefits

**Figure 1:**
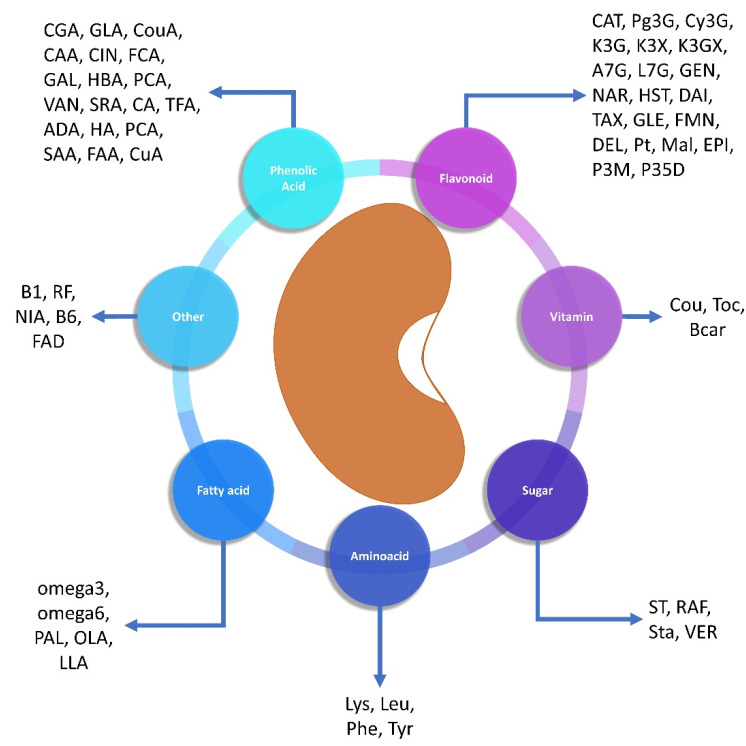
Compounds identified in *Phaseolus vulgaris* L. A7G: apigenin-7-glucoside [51], ADA: aldaric acid [51], B1: thiamine [33], B6: vitamin B6 [33], Bcar: β-carotene [52], CA: kaphtharic acid [53], CAA: caffeic acid [51], CAT: catechin [52], CGA: chlorogenic acid [54], CIN: cinnamic acid [51], Cou: coumetrol [51], CouA: coumaric acid [51], CuA: p-coumaryl aldaric acid [55], Cy3G: cyanidin-3-glucoside [52], DAI: daidzein [51], DEL: delphinidin [51], EPI: epicatechin [56], FAA: feruloyl aldaric acid [51], FAD: folic acid [33], FCA: ferulic acid [51], FMN: formononetin [51], GAL: gallic acid [51], GEN: genistein [51], GLA: syringic acid [54], GLE: glycitein [51], HA: synapic acid [51], HBA: hydroxybenzoic acid [51], HST: hesperetin [51], K3G: kaempferol-3-glucoside [57], K3GX: kaempferol 3-glucosylxilosido [57], K3X: kaempferol 3-xylosylglucoside [57], L7G: luteolin-7-glycoside [51], Leu: leucine [58], LLA: linolenic acid [26], Lys: lysine [58], Lys: lysine [58], Mal: malvidin [51], NAR: naringenin [51], NIA: niacin [33], OLA: oleic acid [59], omega-3: omega-3 [54], omega-3: omega-3 [54], omega-6: omega-6 [54], P35D: pelargonidin 3,5-diglycoside [51], P3M: pelargonium-3-(6″-malonyl)glucoside [51], PAL: Palmitic acid [59], PCA: Protocatechuic acid [51], PCA: Palmitic acid [56], Pg3G: Pelargonidin-3-glucoside [52], Phe: phenylalanine [58], Pt: petunidine [51], RAF: raffinose [60], RF: riboflavin [33], SAA: sinapil aldaric acid [55], SRA: syringic acid [51], ST: starch [54], Sta: Stachyose [60], TAX: taxifolin [51], TFA: transferulic acid [51], Toc: tocopherol [52], Tyr: tyrosine [58], VAN: vanilic acid [51], VER: verbascosa [60]. Source: created by the authors.

Common beans have been highlighted as an almost perfect food due to their high content of protein, fiber, prebiotics, vitamins, and chemically diverse micronutrient composition. They have been shown to protect against oxidative stress, CVD, diabetes mellitus, metabolic syndrome, and many types of cancer [19]. Many compounds have been identified in *P. vulgaris*, such as phenolic acids (chlorogenic acid, syringic acid, caffeic acid), flavonoids (kaempferol, pelargonidin, cyanidin, delphinidin), sugars, fatty acids, and tocopherols, among others [51] (Figure 1).

Dried beans are an enriched source of protein, essential vitamins, minerals, soluble fiber starch, phytochemicals, and are low in fat. Beans have been characterized as a functional food because it has a varied content of bioactive compounds, among which the following stand out enzyme inhibitors, lectins, phytates, oligosaccharides, and phenolic substances that have an effect on the metabolism in humans and animals that consume frequently this food [38,61].

The protein content of beans varies between 16–33%, differences attributed to the environment, crops, years, geographical location, climate, soil conditions, and fertilization [62]. Bean proteins are rich in lysine and poor in methionine and cysteine, being a good complement to cereal proteins, which are deficient in lysine but rich in these sulfur amino acids [30,34]. It ranks as the main source of protein for vegetarians, as well as for low-income age groups throughout the world [63]. This species is used together with cereal to obtain essential amino acids at a low cost [47]. The use of beans rich in protein together with the consumption of cereals offers the best strategy to combat the problem of malnutrition [26,64]. This food strategy is in practice in Latin America, East Africa, and most of Asia [26,27,45].

Beans contain high amounts of glutelin (20–30%) compared to other legumes [65,66]. Glutelins are proteins that are not evenly dispersed over the entire surface of the grain; 80% are practically condensed in the endosperm. Globulins constitute the main fraction in beans, representing between 50–70% of total proteins [26]. The phaseolin is present between 40–50% of the total seed. Prolamine (2–4%) and the set of free amino acids (5–9%) are the other nitrogenous fractions of beans [67].

The amino acid content in common beans has been reported to be variable, although most authors report that the main amino acids are lysine (10–104 mg g^−1^), leucine (14–92 mg g^−1^), and phenylalanine + tyrosine (53–105 mg g^−1^) [63]. Conversely, beans lack the sulfur amino acids, methionine + cysteine (4.0–20 mg g^−1^) [68,69,70]. Amino acids participate in the regulation of metabolism for growth, development, and homeostasis of the body [71]. Some studies show that a diet supplemented with arginine, glutamine, leucine, and cysteine has a positive impact on health [58,72].

Carbohydrates are the main component of beans; they are found in significant quantities between 50–60% of the dry matter [26,73,74]. Among the carbohydrates contained in beans, the one with the highest proportion and availability is starch. Bean starch can be degraded into oligodextrin and glucose by different enzymes such as -and β-amylases [26,75,76]. We also find carbohydrate derivatives such as oligosaccharides [26,77]. The starch, dietary fiber, and non-starch polysaccharides (pectins, gums, hemicelluloses, inulin, fructans, stachyose, and raffinose) present in pinto beans can support the microbiome and/or fermentable products, benefiting the functioning of the gastrointestinal tract [19].

The fat content in *Phaseolus vulgaris* L. was found to be 2.20–5.03% [29]. The main lipid components in beans are phospholipids and triacylglycerols, while minor amounts of diacylglycerols may also be present (hydrocarbons, esters, and hydrocarbons). These lipids can also take the form of phosphatidylcholine, phosphatidylethanolamine, and phosphatidylinositol in beans [26,78]. Butter beans contain high amounts of saturated fatty acids, 28.7 g/100 g [26,79], where we find palmitic and stearic acids.

Polyunsaturated fatty acids represent an important class of lipid compounds present in this species, specifically omega fatty acids (linoleic acid (n-6) and alpha-linolenic acid (n-3) [19]; they comprise 61% of the total fatty acids together with palmitic, oleic [59]. Linolenic acid is dominant among unsaturated fatty acids, constituting 43.1% of the fatty acids in beans [26,80]. It was reported that linoleic and linolenic fatty acids are most of the fat of beans. Oleic and linoleic acids in common beans were determined between 7.8–13.8% and 16.7–25.8%, respectively [29]. Epidemiological studies evidence a favorable effect on the health of patients with coronary heart disease when fed alpha-linolenic acid [19]. Omega fatty acids have a positive effect against obesity, strengthen the immune system and prevent the development of dyslipidemia. In addition to being physiologically and biochemically essential for bodily functions, they also play an important role in the development of healthy tissues [29].

*Phaseolus vulgaris* L. contains a rich variety of phytochemicals with antioxidant activity, such as phenolic acids, flavonoids, flavanols, isoflavones, anthocyanins, and proanthocyanidins [23,26,81]. The anticancer activity of beans has been attributed mainly to their polyphenolic compounds [82,83,84].

Soluble vitamins such as thiamine, riboflavin, niacin, vitamin B6, and folic acid have been identified in common beans [35]. Results indicated the presence of wide variability of macro and micronutrients such as K, Ca, Mg, Zn, Cu and Fe [12]. A higher content of Fe and Ca has been detected in seeds of wild beans compared to those of domesticated local varieties and other improved cultivars [33]. Sangronis and Machado identified phytic acid, tannins, ascorbic acids, thiamine, and minerals as Ca, Mg, Zn, Fe and Cu [85]. The essential minerals, Se, Fe, and Zn, are of great importance for human health. It has been identified that Se strengthens the immune system and reduces the risk of cancer [29]. Anemia, for example, is caused by Fe deficiency; it has been described that there are more anemic people in developing countries than in Europe and the United States [29]. Paredes et al., 2019, indicated the presence of wide variability of macro- and micronutrients: N, Fe, and Zn in bean genotypes previously classified as Chile race. These showed an adequate level of proteins, Fe and Zn; additionally, they did not show significant differences with genotypes of other races. The content of N was positively related with the proteins, P, Cu and Zn, while the content of Fe was correlated with Mn, and the content of Ca and Zn was correlated with the content of N, P, Cu, and S [38].

The levels of B vitamins present are considerably high. One cup of beans (225 g cooked) provides approximately 74% folic acid, which has multiple biological functions, deoxyribonucleic acid synthesis, repair, and methylation, as well as acting as a cofactor in many reactions. Folic acid is necessary to prevent anemia in children and adults. Dried beans contain vitamins A and C that represent 3% to 8% of the daily reference intake. One cup of beans provides adequate amounts of vitamin B6, E, K, and thiamine. Vitamin E is a powerful antioxidant and anti-inflammatory agent. Intake of vitamin K is widely associated with bone health and anti-cancer properties [19]. The consumption of approximately ½ cup of dry beans or peas is related to a higher intake of fiber, protein, folic acid, Zn, Fe, and Mg, while conversely shows a lower intake of saturated fat and total fat [86].

It can be said that the consumption of beans is associated with favorable effects on health, such as cholesterol level reduction and coronary heart disease, cancer diseases, reduction of diabetes and obesity, antioxidant power, antimutagenic and antiproliferative, among many others [38,41,83,87,88,89].

## 4. Role of Beans in CVD

### 4.1. Effect on Hemostasis and Platelet Aggregation

The complex pathophysiological process involved in CVD includes the participation of platelets; these have a main role during thrombosis and progression of atherosclerosis [90]. Food supplements and/or nutraceuticals have become attractive alternatives to reduce cardiovascular events [91].

The methanolic extract of *Phaseolus vulgaris* L. had been considered relevant by its antiplatelet effect, especially the ability to suppress platelet secretion, using the proposed mechanism of protein kinase A (PKA) modulation and the inhibition of AKT phosphorylation [92].

It is hypothesized that some flavonoids (kaempferol, epicatechin, delphinidin, cyanidin) can inhibit the platelet function by suppressing the platelet aggregation, calcium mobilization, integrin modulation, granule secretion, and thrombus formation, using as an example the pharmacological action of nobiletin [93].

Furthermore, other proteins can increase the activation of platelets, as is the case of lectins; these are proteins sometimes referred to as an antinutrient for decreasing the body’s ability to absorb nutrients, but this review will be focusing on the lectins-induced stimulation of fresh platelets [94].

These lectins have shown effect through phospholipase C (PLC) ƴ 2 activations, using the Src/Syk and PI3K/BTK pathways, but also an increase in the reactive oxygen species (ROS), as well as superoxide anion formation and lipid peroxidation by working as an uncoupling agent with the consequent increase in oxygen consumption and decrease in adenosine triphosphate (ATP) formation [95,96]. This activation was completely inhibited by the use of penicillin G (12.5 mM) and cephalothin (12.5 mM) [97].

Another interesting discovery is the *Phaseolus vulgaris* L. agglutinin production of nitric oxide (NO), regulated by the Ca^2+^/calmodulin kinase/AMPK pathway in a time and dose-dependent manner. This process is dependent on the eNOS phosphorylation involving the eNOS/NO/cGMP/PKG pathway [95,98]; the NO production by the beans agglutinin can reduce the platelet aggregation, explaining the lower platelet activation compared with agglutinin from whole grain [99].

Many components from *Phaseolus* can help with the regulation of platelet aggregation as glycine by impeding the calcium influx [100], arginine by enhancing the nitric oxide activity in hypercholesterolemic patients [101], or anthocyanin by inhibiting the platelet-monocyte and platelet–endothelial interaction [102].

Derivatives of alpha-linoleic acid can inhibit platelet aggregation and inflammation, which has been linked to the prevention of CVD, hypertension, type 2 diabetes, chronic obstructive pulmonary disease, among others [19]. Especially, the role of omega3 and omega6 has been discussed many times for his role in platelet aggregation; many papers report the inhibitory effect of omega3 in ADP-dependent platelet aggregation [103,104], with a noticeable effect in healthy patients, unlike CVD patients who had a low increase in lag time [105]. Meanwhile, high omega 6 levels have been related to pro-inflammatory and pro-aggregatory phenotypes [106], by increasing the susceptibility of LDL to oxidate and therefore increasing the TXA2 production [107].

### 4.2. Effect on the Endothelium

The ingestion of inadequately cooked beans can result in severe glycemic index tract distress; the proposed mechanism of damage prevents the repair of the epithelial cell surface disruptions, resulting in necrotic cell death. The lectins are known to inhibit the exocytosis event required to repair the plasma membrane, and that is the mechanism used to maintain and accumulate the damage in the GI tract [108].

As we discussed, the common bean is cultivated worldwide and used as a nutraceutical food, when cooking properly, but is not the only process used to gain nutritional value from these pulses. The common bean hydrolysate had reported many effects from angiotensin-converting enzyme inhibitor to antioxidant, antimicrobial, and even tumor cell inhibitor. The bioactive potential of peptides present in the indigestible fraction of common beans that protect cells from oxidative stress and inhibit the angiotensin-I converting enzyme by interacting with its catalytic cavity independently of its antioxidant capacity was demonstrated [109].

Gomes et al. explained the hydrolysate capacity to modulate lipid metabolism and prevent endothelial dysfunction in BALB/c mice; they also showed hypocholesterolemic activity helping to reduce inflammation, oxidative stress, and endothelial dysfunction [110]. *Phaseolus vulgaris* L. agglutinin (PHA) evidenced specific cytoplasmic staining of macrophages in rabbit vessels, monkeys, and human tissues (atherosclerotic arteries obtained in surgery). When analyzing the morphometric comparisons between PHA staining of the lesion and acid lipase as a macrophage marker, similar results were obtained. In this context, they concluded that PHA is an excellent experimental marker to differentiate and quantify macrophages in fixed and human atherosclerotic lesions [111]. The use of hydrolysates of *Phaseolus vulgaris* shows an interesting effect in mice, from the modulation of the lipid profile to the increase in e-NOS expression [110]; this effect can be explained by the effect of the compounds found in the bean, upon endothelial cells. This is the case of n-3 PUFAs such as omega3 that, in trials, have shown prevention of endothelial dysfunction [112], or of amino acids such as lysine, leucine, serine and glutamine that work as modulators of NO production [113].

### 4.3. Effect on Inflammation

Some studies have focused on evaluating the effect of different plants with beneficial effects on pro-inflammatory mechanisms, mainly to reduce cardiovascular risk factors [114]. Macrophages are the main source of pro-inflammatory cytokines and can be used as markers of chronic inflammation, tumor necrosis factor α (TNF-α), interleukins (IL), and prostaglandins E-2 (PGE-2), among others. TNF-α plays a fundamental role in the expansion of the inflammatory process since it induces the production of IL-1β, among other pro-inflammatory cytokines [115], and increases PGE-2 [116,117].

Peroxisome Proliferator-Activated Receptors (PPARs) are transcription factors that belong to the superfamily of ligand-activated nuclear receptors, which mainly regulate lipid metabolism [118]. PPAR-α ligands are known to have anti-inflammatory effects in various cells through apoptosis in cytokine-activated macrophages, inhibiting NFκB signaling [118,119]. It has been described that the enzymatic hydrolysis of beans produces protein hydrolysates with anti-inflammatory activity [120] that could counteract the chronic inflammatory process initiated by human macrophages [117]. Research highlights the effectiveness of total digested proteins and peptides from bean seeds against adipogenic complications and inflammation [121]. Hydrolysate protein from this legume has been shown to decrease inflammation in adult male mice fed an atherogenic diet for nine weeks [110].

Phaseolin is the main globulin reserve in bean seeds [67]. This protein is a potential therapeutic candidate for the management of inflammation. Phaseolin inhibits nitric oxide production; inducible nitric oxide synthase expression also suppresses pro-inflammatory mediators such as cyclooxygenase 2 (COX-2), interleukin-1β (IL-1β), tumor necrosis factor α (TNF-α), among others [122].

Oseguera et al. evaluated the antioxidant capacity of protein hydrolysates (rich in bioactive peptides derived from phaseolin) from the Negro 8025 and Pinto Durango varieties of *Phaseolus vulgaris* L. and determined their effect on the markers of inflammation in RAW 264.7 macrophages induced by lipopolysaccharides. Durango Pinto bean alcalase hydrolysates at 120 min inhibited inflammation (inhibition of cyclooxygenase (COX)-2 expression, prostaglandin E_2_ production, inducible nitric oxide synthase (NOS) expression, and NO production) to a greater extent than black beans. Additionally, the hydrolysates inhibited the transactivation of NF-κB and the nuclear translocation of the p65 subunit of NF-κB [120].

Kim et al., 2016, studied the effects of adzuki beans on lipid accumulation and inflammation mediated by oxidative stress in male C57BL mice induced by a diet high in cholesterol and fat for 6 weeks. The results suggested that adzuki beans decrease lipid accumulation and inflammation induced by oxidative stress, by a mechanism of suppression of hepatic messenger RNA expression of lipogenic and inflammatory mediators. This effect could be associated with the rich anthocyanin, catechin, and saponin content of adzuki beans [123].

The effect of whole wheat flour and bean protein hydrolysate from the common bean variety Carioca on inflammation and oxidative stress was studied in BALB mice fed a diet high in fat and cholesterol. Animals fed whole bean meals showed less weight gain, higher levels of alanine aminotransferase, and low-density lipoprotein cholesterol than animals fed bean protein hydrolysate. The expression of PPAR-α was lower in the groups fed with bean protein hydrolysate and bean flour. These results could be associated with the increase in inflammation generated in diet-induced obesity since a short period was sufficient to decrease the inflammatory marker (PPAR). The positive effect on inflammation is attributable to phenolic compounds such as catechin and kaempferol present in bean flour, while in the protein hydrolysate; it is attributed to biologically active peptides and proteins such as phytohemagglutinin, alpha and beta phaseolin, alpha-amylase 1 inhibitor, and alpha-amylase 2 inhibitors [119,124].

Another study refers to how postharvest storage time influences the inflammation of Carioca, Madreperola, and Pontal beans, stored (0, 3, and 6 months), cooked, and subjected to simulated gastrointestinal digestion with pepsin–pancreatin. The study was conducted in human THP-1 macrophage-like cells. The commercial storage time did not affect the protein concentration, the degree of hydrolysis, the hydropathic, or the antioxidant capacity. All hydrolysates reduced TNF-α by about 30%. The Madreperola hydrolysates decreased IL-1β and PGE-2. Carioca beans inhibited inflammation due to their content of bioactive peptides and phenolic compounds, and it was shown that the commercial storage time did not affect the physicochemical or biological properties [117].

Studies have shown that the antioxidant and anti-inflammatory activities of bean extracts are associated with polyphenols present capable of inhibiting COX and lipoxygenase (LOX). Acetone extract made from black bean peel exhibited strong inhibitory effects of COX-1 (IC_50_ = 1.2 μg/mL) and COX-2 (IC_50_ = 38 μg/mL), while the aqueous extracts were stronger inhibitors of lipoxygenase, 15-LOX, versus the acetone extracts. The COX and LOX inhibitory activities of aqueous extracts such as acetone suggest that the use of bean shells in food may protect against some diseases associated with chronic inflammation [125]. People who consume beans and whole grains have been found to have a longer life expectancy and lower burden of chronic diseases, including obesity, CVD, diabetes, and cancer [126], which are characterized by having a strong chronic inflammatory component [127].

### 4.4. Effect on Metabolic Syndrome

Metabolic syndrome (METS) is a simultaneous group of metabolic disorders that includes central obesity (abdomen), insulin resistance, hypertension, glucose intolerance, and dyslipidemia [128], which increases the risk of CVD. It is estimated that it affects almost 35% of the US adult population, and its prevalence increases with age [129].

A healthy lifestyle, improving eating habits, and physical activity, are the therapeutic recommendations for the treatment and management of METS, but a gold standard dietary pattern for its management has not yet been proposed [130]. In this sense, many researchers have pointed out that a diet high in unsaturated fats, (olive oil), together with the consumption of legumes, cereals (whole grains), fruits, vegetables, nuts, fish, and low-fat dairy products, can prevent and delay the development of METS and prevent CVD [131].

One of the main causes of the development of this chronic syndrome is an imbalance between caloric consumption and expenditure. METS is associated with excessive activity of glucose metabolism enzymes and inflammatory processes [132]. Thus, a diet with low glycemic index products, such as *Phaseolus vulgaris* L., slows down the absorption of carbohydrates due to the inhibition of alpha-amylase and glucosidase enzymes, been proven in clinical trials [133].

Products that slow the absorption of carbohydrates by inhibiting the enzymes responsible for their digestion have been described as a powerful alternative to achieving a low-glycemic diet. These products include alpha-amylase and glucosidase inhibitors, which can reduce the risk of diabetes and heart disease and its complications. The common white bean (*Phaseolus vulgaris* L.) inhibits alpha-amylase by the action of the alpha-amylase inhibitor protein (αAI), which has been characterized and demonstrated in various clinical studies, demonstrating the ability of beans to cause weight loss (doses between 500 to 3000 mg per day). Conversely, the ability of this legume to reduce the postprandial peak in blood glucose levels depending on the dose has also been pointed out [133,134]. Common beans have three isoforms of alpha-amylase inhibitors (alpha-A1, alpha-A12, alpha-AIL). The alpha-AI isoform has anti-amylase activity in humans. This enzyme is only found in the embryonic axes and cotyledons of the plant seed. The alpha-amylase inhibitor prevents starch assimilation by completely blocking access to the active site of the enzyme. Some factors that affect the activity of the alpha-AI isoform inhibitor are pH, temperature, incubation time, and the presence of particular ions. Several authors have pointed out that the common bean reduces the rate of carbohydrate absorption, thus reducing the glycemic index of foods, as well as weight loss when consumed at the same time with carbohydrate-containing meals [133].

The consumption of legumes such as *Phaseolus vulgaris* L. provides bioactive molecules with an effect on obesity and metabolic syndrome, mainly due to a decrease in weight and triglyceride levels, although more quality trials must be performed to establish clinical efficacy [135]. In this sense, overweight individuals who received *Phaseolus vulgaris* L. extract had a significantly greater reduction in body weight index, fat mass, adipose tissue thickness, and anthropometric measurements of waist, hip, and thigh compared to the placebo group. The authors point out that this effect is based on the activity of αAI described in the extracts of *Phaseolus vulgaris* [136]; furthermore, the daily consumption of baked beans (*Phaseolus vulgaris* L.) for 14 days as part of a regular diet significantly decreased the mean total plasma cholesterol level of the volunteers: from 5.1 to 4.5 mmol/L (*p* < 0.02) [137]. This is correlated with the effect of dry beans, where it was identified that they reduce serum lipid concentrations in healthy and hyperlipidemic subjects, specifically serum cholesterol and triglyceride concentrations by 10.4% (*p* < 0.001) and 10.8% (*p* < 0.025), respectively, along with reducing body weight, despite constant energy intake, contributing to the management of hyperlipidemia present in METS due to its high content of soluble fiber, which alters the absorption of lipids in the intestine, affecting the synthesis of cholesterol to hepatic level [138].

In a clinical trial with 12 adults diagnosed with METS who ate one of three meals: black beans (BB), combined fiber (FM), and combined antioxidant capacity (AM), it was found that in the group that consumed black beans, postprandial insulinemia was lower after the meal compared to the other groups (*p* < 0.0001), and there was an improvement in plasma antioxidant capacity (*p* = 0.002), which could be explained by the fiber content of beans [139]. Similar effects were seen in healthy individuals, where consumption of *Phaseolus vulgaris* L. extract reduced postprandial glucose, insulin, and increased satiety [140]. Finally, 12 volunteers with METS were given in three different meals: no added fiber (control (NF), extrinsic or added fiber (AF), or whole black beans as a source of intrinsic fiber (BN). The BN meal produced a significant reduction in comparison with controls (*p* < 0.0001), showing beneficial effects in patients with METS [141]. In the context of animal models, in METS-induced male C57BL/6 mice, *Phaseolus vulgaris* L. extract reduced body weight and effectively lowered blood glucose, triglycerides, and cholesterol. At the same time, histological analysis of the aorta showed protection against the development of fatty streaks in the muscle layers. The authors conclude that the mechanism of action is due to the presence of αAI and alpha-glucosidase inhibitors [142]. In another investigation, treatment with a combination of *Phaseolus vulgaris* L. and *Cynara scolymus* extracts reduced food intake and blood glucose in rats [143].

Proteins are abundant components in beans. The positive effect on blood pressure reduction of bean protein hydrolysates has been reported in hypertensive rats, which is attributed to the ability of peptides to inhibit angiotensin-converting enzyme (ACE) [110,144]. Glutelin hydrolysates show a potent ACE inhibitory activity of around 80.24%. The results show that glutelin could be an effective hypertensive in ACE [145]. Conversely, the administration of a black bean protein hydrolysate at a concentration of 200 mg/kg showed a hypoglycemic effect in rats [110,146]. Studies have highlighted that starch-enriched diets lower cholesterol levels, improving dyslipidemia and body composition. A double-blind, placebo-controlled crossover intervention showed that individuals who consumed a diet rich in starch for 12 weeks show favorable results for the promotion of these diets in public cardiometabolic health [147]. Another study examined the effect of starch on hypolipidemic actions, blood glucose, insulin levels, and humoral immune responses in healthy, overweight subjects who were fed 24 g/day of resistant cornstarch or regular cornstarch for 21 days. Reducing effects of total serum cholesterol and serum LDL cholesterol were evidenced. These results suggest that starch supplementation improves blood lipid profile and controls blood glucose levels in healthy overweight subjects [148].

Many studies suggest the effect of linoleic acid on obesity, cancer, atherosclerosis, among other health benefits. Linoleic acid has been shown to promote fat loss in rodent models [149]. Initial studies in male and female mice showed that a mixed diet supplemented with conjugated linoleic acid promotes fat loss by 60% for 30 days; this effect was attributed to increased lipolysis and fat oxidation [149,150]. Recently, it has been recognized that supplementation with this acid reduces fat stores, and dramatically decreases circulating adiponectin levels in mice [149,151].

Evidence suggests that the consumption of derivatives of *Phaseolus vulgaris* L. reduces food intake, body weight, lipid deposition, and blood glucose in rats due to the inhibition of α-amylase, reducing carbohydrate metabolism [152]. Together, these data in animal models and clinical trials demonstrate the potential effect of *Phaseolus vulgaris* L. to treat obesity and METS, consecutively decreasing the development of thrombotic events.

### 4.5. Effect of Beans on Atherosclerosis

Studies have shown that diet attenuates atherosclerosis, the mechanisms of which are related to less atherogenic dyslipidemia, relief of intestinal dysbiosis, and suppressed inflammation [153]. The atherosclerotic process is established from the increase of pro-atherogenic and pro-inflammatory mediators that favor plaque formation and progressive stenosis [154,155]. The initial step of atherosclerosis is associated whit high levels of low-density lipoproteins (LDL), oxidation of LDL, and recruitment of monocytes [155,156]. The accumulation of cholesterol-laden macrophage foam cells is a key feature of atherosclerotic lesions. Cholesterol can enter macrophages through various pathways and induce the transformation of macrophages into foam cells [157].

Studies have shown that beans can improve lipid profiles associated with the development of atherosclerotic lesions and the prevalence of CVD. Consuming beans lower cholesterol without affecting serum triglycerides, VLDL cholesterol, or blood glucose [158]. Oxidized LDL (ox-LDL) and its interaction with the ox-LDL lectin receptor (LOX-1) determine the progression of atherosclerosis. Peptides from carioca beans have shown antiatherosclerotic properties comparable to simvastatin, through inhibition of LOX-1, MMP-9, and ICAM-1 and inhibition of 10 cytokines related to the atherosclerotic process (128). Research shows that chia, a variety of beans, is considered a good source of dietary fiber, protein, antioxidants, and bioactive lipids [159,160]. In recent years, chia seeds have gained great importance due to their high alpha-linolenic acid content (68%) and their relationship to human health and nutrition [161].

Various studies have indicated that this compound has cardioprotective properties by affecting specific biomarkers (lactate dehydrogenase; LDH) [162]. The background shows that conjugated linoleic acid has the potential to inhibit cholesterol-induced atherosclerosis in rabbits and hamsters, respectively [163]. Conjugated linoleic acid inhibits experimentally induced atherosclerosis in rabbits fed an atherogenic diet. A reduction in pre-established atheromatous lesions was evidenced [164]. Conjugated linoleic acid reduced early aortic atherosclerosis to a greater extent than linoleic acid in a hypercholesterolemic hamster population. These effects may be related to changes in the oxidative susceptibility of LDL in hypercholesterolemic hamsters [165].

The intake of dietary fiber has been associated with an inhibition of the development of atherosclerosis in animal models [88], while soluble fiber reduces serum cholesterol and LDL cholesterol concentrations [88,166]. In general, proteins of animal origin are more cholesterolemias and atherogenic than proteins of plant origin [167]. Research has highlighted that the oral administration of peptides synthesized from amino acids reduces atherosclerosis independently of plasma cholesterol in a group of mice, thus improving the capacity of high-density lipoproteins (HDL) in the study population [168]. Specific studies with different varieties of beans have shown that the consumption of this legume reduces 10% of the cholesterol levels of normal young men after ingestion of 450 g/d of canned baked beans compared to the control group [137]. A 10% reduction in serum cholesterol levels was also reported in hyperlipidemic men fed 120–162 g/d of pinto beans [138].

Celleno et al. showed that overweight subjects who consumed a dietary formula with *Phaseolus vulgaris* L. extract as the main ingredient, significantly decrease body fat due to the interference caused by this legume in the digestion of carbohydrates, thus contributing to the prevention of atherosclerosis by reducing fats in organs and tissues [136]. *Phaseolus vulgaris* L. provides micronutrients, particularly folic acid and magnesium, and its high content of fiber, sulfur amino acids, tannins, phytoestrogens, and non-essential amino acids have been linked to the prevention of atherosclerotic lesions. The prevention of atherosclerosis is a powerful tool in the prevention of cardiovascular events, as this silent pathology is responsible for about half of deaths from heart disease [19,169].

### 4.6. Other Studies Related to Cardioprotective Role

The intake of *Phaseolus vulgaris* L. has been decreasing during the last decades, which has coincided with an increase in the incidence of chronic diseases such as obesity and CVD [170]. Epidemiological studies show that the consumption of legumes is inversely associated with the risk of coronary heart disease, type II diabetes mellitus, and obesity; specifically, the intake of beans is associated with the reduction of risk factors by regulating glycemia and normalizing the lipid profile in the blood [171]. Researchers point out that it is possible to reduce the incidence of coronary heart disease with diet [172] and that the consumption of beans reduces the risk of ischemic heart disease and CVD [170].

Some studies show that bioactive molecules such as the phenolic compounds of *Phaseolus vulgaris* L. are associated with a protective effect against CVD, diabetes, and cancer; due to their antioxidant and anti-inflammatory properties [173,174]. Those who have a diet that includes *Phaseolus vulgaris* L. bean species have a lower prevalence of diabetes, CVD, and cancer [175]. The cardioprotective effect of beans is associated with its content of kaempferol and catechin, which lower cholesterol levels, use HDL levels, and inhibit lipid peroxidation [176]. This effect was reflected in a clinical trial, where the consumption of a cup of beans, decreased LDL levels after 6 hours when compared with the control (consumption of rice). In addition, a positive effect was generated on the tension properties of the blood vessels, favoring vascular function [177].

In an in vitro study, the extract of hardened common bean residue (composed of a peptide fraction <3 kDa (PV3)) reduced ROS levels and increased NO in the endothelium, favoring vasodilation and showing a therapeutic potential [178]. Along the same lines, bean extracts significantly decreased the percentage of platelet aggregation in platelet-rich plasma, induced by adenosine 5′-diphosphate (ADP) and arachidonic acid, along with decreasing activation markers expressed in the platelet membrane (P-selectin). This supports the hypothesis that common beans reduce the risk of CVD by reducing platelet activation processes that initiate thrombus formation [92].

In animal models, Sprague–Dawley rats and a diet-induced obesity model in C57Bl/6 mice were used to evaluate the effect of cooked dry beans; in both animal species, short-term feeding decreased total plasma cholesterol and LDL cholesterol after 14 days [179]. In this context, the incorporation of extruded beans into the diet of obesogenic rats reduced the levels of cholesterol and liver triglycerides (*p* < 0.001) and low-density lipoproteins (LDL; *p* < 0.01), favoring metabolism of lipids [180]. These results together show a cardioprotective effect of *Phaseolus vulgaris* L.

Additionally, studies have shown that a low glycemic index diet reduces the risk of cardiovascular events. Nourí et al. showed that a population of around 5000 subjects who consumed legumes as the main food decreased the occurrence of CVD by around 34%, including fatal and non-fatal myocardial infarction, unstable angina, fatal and non-fatal stroke, and death sudden cardiac arrest. This study indicated an inverse relationship between legume intake and the risk of CVD in elderly people [63].

The intake of fatty acids, dietary fiber, and antioxidant compounds reduce the risk of heart disease [88,181,182]. Epidemiological studies suggest that the high intake of complex carbohydrates and dietary fiber is directly related to the decrease in coronary artery disease [88,183]. Beans provide all these nutrients with cardioprotective properties to our diet.

Additionally, diets enriched with polyunsaturated fatty acids such as omega-3 show promise in the primary and secondary prevention of CVD. Studies have been encouraging, showing positive results for in vivo trials in preventing myocardial infarction and heart failure [184]. Omega-3 can prevent fatal arrhythmias through the inhibition of sodium and calcium channels [184,185]. One study found an inverse association between the intake of baked or roasted fish and heart failure in 4738 men and women [184,186]. Another 13-year investigation revealed the beneficial effects in preventing CVD mortality of a diet rich in omega-3 of a Japanese population [184,187]. Conversely, dietary supplementation with B vitamins or omega-3 fatty acids, or both, could prevent major cardiovascular events in patients with a history of ischemic heart disease or stroke [188].

Table 1 shows the compounds identified in *P. vulgaris* with a history of cardioprotective potential. This analysis could promote the consumption of this legume considering that it could have a beneficial effect on several CVD risk factors. It has been shown that certain components of the diet can reduce blood platelet activation and have an important influence on the prophylaxis and treatment of cardiovascular disorders [2].

## 5. In Silico Assays of Bioactive Compounds from *Phaseolus vulgaris* L.

The development of structural elucidation by X-ray crystallography and bioinformatics has made it possible to identify and/or predict target–metabolite interactions. In this field, some studies have been carried out to determine the activity of metabolites present in *Phaseolus vulgaris* L. on different targets.

**Figure 2:**
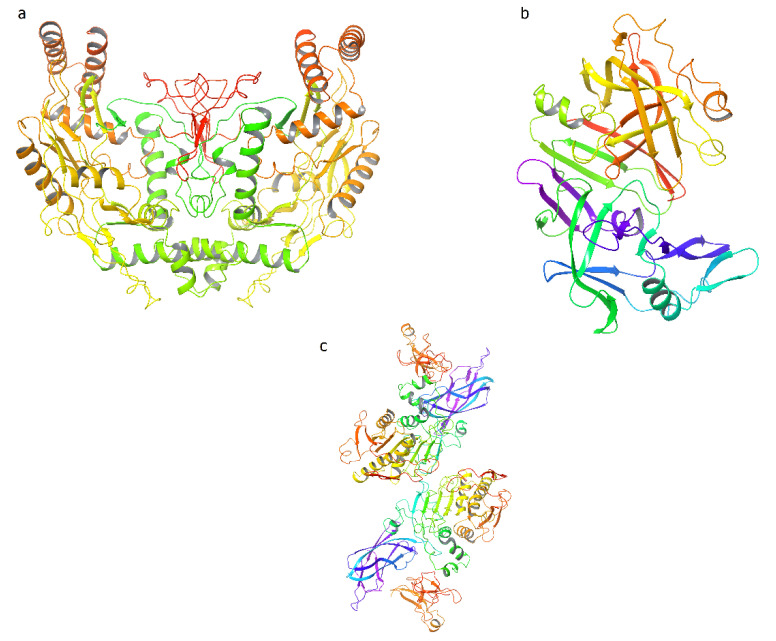
Targets studied about *Phaseolus vulgaris*. (**a**) NOS protein (PDB:ID 3NLE) [237]. (**b**) Porcine gastric mucosa (PDB code: 5PEP [240]. (**c**) Porcine pancreatic lipase (PDB code: 1 ETH) [241]. Source: adapted images according to PDB ID.

Petchiammal C. and Waheeta Hopper, 2011, performed molecular docking assays using 28 bioactive compounds from *Phaseolus vulgaris* extracted from Dr. Duke’s database (https://phytochem.nal.usda.gov/phytochem/plants/show/1470, accessed on 29 November 2021). These metabolites were evaluated as agonists of NOS, a therapeutic target that is found modulating the high pathological concentration of NO (antioxidant activity). The crystal structure utilized was the NOS heme domain (PDB: ID 3NLE) [237] (Figure 2a). Twenty-eight compounds from *Phaseolus vulgaris* were tested; among them, allantoic acid was found to have the best interaction with the residues present in the active site (Arg367) and the heme subunit of the NOS enzyme (Figure 2). To corroborate this, it was compared with the ligand present in the crystal. It was observed that the co-crystallized ligand also (allantoic acid) shows a better binding affinity toward the heme domain and the reported residues of the active site.

In silico analysis of allantoic acid from *Phaseolus vulgaris* L. suggests that this compound could have antioxidant activity by acting as an inhibitor of the NOS enzyme [238].

Peptides from *Phaseolus vulgaris* cv. Pinto has been evaluated as bioactive peptides in the n regulation of physiological functions: protease activation, lipase inhibition, and bile acid-binding activities. About nine peptides have been identified in *Phaseolus vulgaris* with protease activity and pancreatic lipase activity [239]. Ngoh et al., 2017, used the crystallographic structures of porcine gastric mucosa pepsin (PDB code: 5 PEP) [240] (Figure 2b), and porcine pancreatic lipase (PDB code: 1 ETH) (Figure 2c). In vitro and in silico analyses were able to demonstrate that five isolated peptides from *Phaseolus vulgaris* cv Pinto had a protease capacity, inhibiting the activity of pancreatic lipase and binding to bile acids. These results were able to demonstrate the efficacy of the peptides efficacy of *Phaseolus vulgaris* cv Pinto as therapeutic agents in the treatment and prevention of cardiovascular diseases (i.e., obesity, hyperlipidemia and hypercholesterolemia).

**Figure 3:**
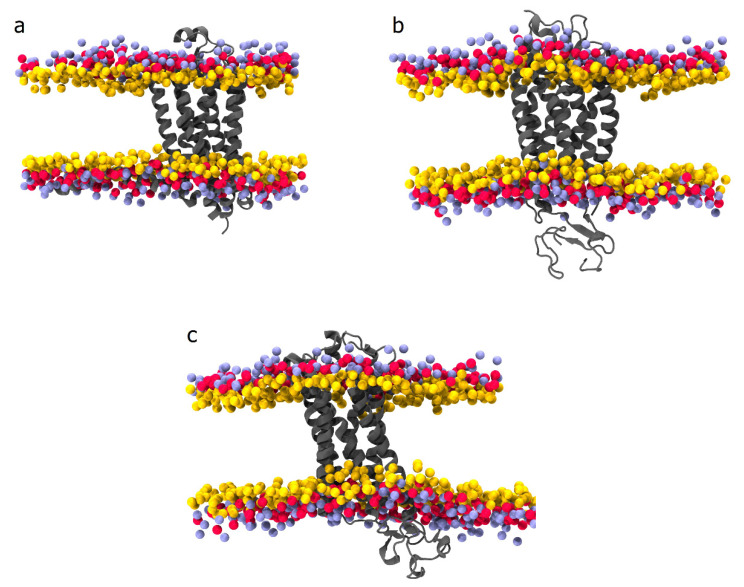
Therapeutic targets associated with CVD. (**a**) Adenosine A2A receptor (PDB ID: 2YDV) [242]. (**b**) P2Y1 receptor (PDB ID:4XNV) [243]. (**c**) Thromboxane receptor TxA2 (PDB ID: 6IIU) [244]. Source: all images were obtained from MemProtMD database according to the PDB ID [247].

Important CVD-associated targets have been crystallized, among these are adenosine A2A receptor (PDB ID: 2YDV) [242] (Figure 3a), P2Y1 receptor (PDB ID:4XNV) [243] (Figure 3b), thromboxane receptor (TXA2) (PDB ID: 6IIU) [244] (Figure 3c), and protein kinase C (PDB ID: 2I0E and 1A25) [245,246].

The adenosine A2A receptor is a G protein-coupled receptor associated with numerous therapeutic applications. In platelets, activation of adenosine A2A receptors (Gs-coupled receptor) is responsible for antiplatelet activity through increase intraplatelet cAMP levels [194,242,248].

The P2Y12 receptor (P2Y12R), one of eight members of the P2YR family expressed in humans, has been described as a relevant pharmacological target because its inhibition decreases platelet aggregation [249].

TXA2 receptor is expressed in platelets, inflammatory cells, the vascular wall, and atherosclerotic plaques [250]. Since the TXA2 receptor plays a vital role in cardiovascular homeostasis, it is considered a vital drug target for CVD [251].

The protein kinase C (PKC) family is a central regulator in platelet activation processes: granule secretion, integrin activation, aggregation, spreading, and procoagulant activity [252].

Future in silico research should focus on the analysis of CVD target crystallographic structures and the identification of metabolites present in *Phaseolus vulgaris* L. to obtain information relevant to the binding mode and the determination of a possible mechanism of action.

## 6. Conclusions

**Figure 4:**
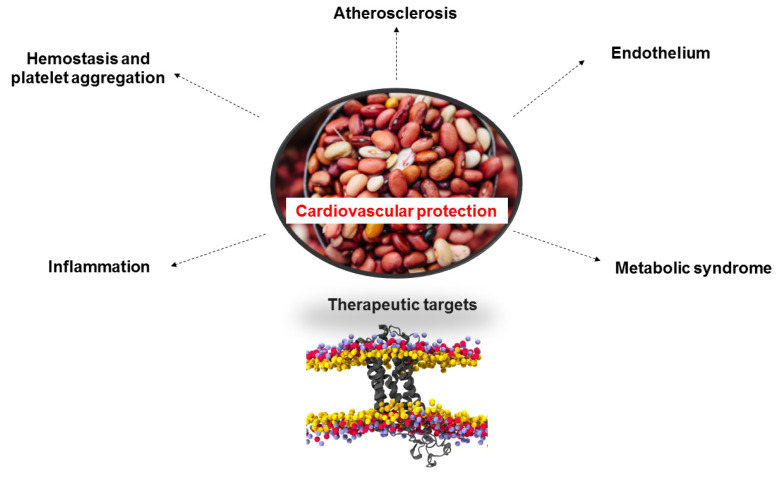
Cardioprotective Effects of *Phaseolus vulgaris* L. Source: created by the authors.

Diet is an important tool to maintain good cardiovascular health and thus reduce mortality and morbidity rates worldwide. This review has shown that the common bean is the food legume with the highest nutritional value, which has caused a greater demand for this product in low-income countries but also recently increased consumption in developed countries, which seek to guide their diet to a healthy style. It was shown that there is a positive correlation between the consumption of beans and the prevention of cardiovascular events (Figure 4). This legume has bioactive compounds (phenolic acids, flavonoids, fatty acids) that give it a unique value as a protector against the endothelium, inflammation, atherosclerosis, metabolic syndrome, among other pathologies of great impact on the population. The fundamental mechanisms on which this product influences have been related mainly to the inhibition of lipid peroxidation and lowering of cholesterol and blood glucose levels, among others. We recommend frequent consumption of beans because they can be used to improve nutritional wellbeing and prevent the prevalence of CVD.

## Figures and Tables

**Table 1:** Cardioprotective studies of bioactive compounds from *Phaseolus vulgaris* L.

Compound	Classification	Mechanisms
Coumaric acid	Phenolic acid	Reduces ADP-induced platelet aggregation in vitro. Modifies platelet function, measured with PFA-100 (500 and 1 µM). Mechanism: inhibits the AA cascade, reducing TxA2 production and the generation of prostaglandin E_2_ induced by lipopolysaccharides (IC_50_: 371 and 126 μM) [189]. Inhibits ADP-induced platelet aggregation in vivo without affecting blood coagulation. Mechanism: associated with a marked increase in plasma antioxidant activity, measured as plasma iron-reducing capacity, and with a reduction in TxA2 production [189].
		Reduces apoptosis in vivo in rats with isoproterenol-induced myocardial infarction by inhibiting oxidative stress (2.4 and 8 mg/kg). Mechanism: protective effect due to anti-lipid peroxidative, antioxidant, and anti-apoptotic properties [190].
		Inhibits in vitro TRAP-induced surface P-selectin expression (100 μmol/L) [191].
		Inhibits in vivo LDL oxidation and decreases MDA production, causing a decrease in atherosclerosis (100 mg/kg) [192].
		Protects the heart in vivo against DOX-induced oxidative stress (100 mg/kg) [193].
Chlorogenic acid	Phenolic acid	Inhibits in vitro platelet secretion and aggregation induced by ADP, collagen, AA, and TRAP-6. Decreases platelet adhesion, aggregation, and platelet–leukocyte interactions under flow conditions. Inhibits platelet inflammatory mediators (P-selectin, sCD40L, CCL5, and IL-1β) and increases intra-platelet cAMP levels by activation of PKA (0.1 to 1 mmol/L). Mechanism: adenylate cyclase/cAMP/PKA receptor signaling pathway [194].
		Inhibits collagen-induced platelet aggregation and suppresses TxA2 production, associated with COX-1 inhibition in platelet microsomes that have activity on cytochrome c reductase. Increases the formation of cAMP and cGMP and intracellular Ca^2+^ (10, 30, and 50 mmol/L). Mechanism: reduction of TxA2 and increased levels of cAMP and cGMP [195].
Ferulic acid	Phenolic acid	Inhibits in vitro the expression of P-selectin on the surface induced by TRAP (100 μmol/L). Inhibits platelet aggregation induced with collagen 3.5 μg/mL (100 μmol/L) [191].
		Inhibits in vitro platelet aggregation stimulated with ADP 8 μmol/L and collagen 1.5 μg/mL (0.5 mmol/L) [196].
		Protects in vivo death produced by pulmonary thrombosis and prolongs tail bleeding time in mice and rats (20, 40, and 80 mg/kg for mice, or 10, 20, and 40 mg/kg for rats). Inhibits in vitro platelet aggregation induced by ADP, thrombin, collagen, and AA. It decreases the mobilization of intracellular Ca^2+^ and the production of TxA2. Increases levels of cAMP and cGMP and phosphoprotein stimulated by VASP while decreasing phospho-MAPK and PDE (200 mM). Mechanism: activation of cAMP and cGMP signaling [197].
Synapic acid	Phenolic acid	Inhibits in vitro platelet aggregation and coagulation with antithrombotic effect (0.25 mg/mL, IC_50_: 1.03 mM) [198].
Protocatechic acid	Phenolic acid	Decreases in vitro SIPA and attenuates platelet activation, intracellular Ca^2+^ mobilization, granule secretion, and adhesion receptor expression (10–25 µM). Mechanism: blockade of the binding of VWF to activated GPIb, primary and initial event for the performance of SIPA [199].
		Reduces myocardial infarction size, serum TNF-α levels, and platelet aggregation in vitro. Inhibits apoptosis and caspase-3 expression and positively regulates phosphorylated Akt expression in cardiomyocytes subjected to injury by H/R (250–500 mg/kg). Mechanism: provides protection against MI/R injury, inflammatory response, platelet aggregation, and apoptosis of cardiomyocytes [200].
		Inhibits platelet apoptosis induced by decreased dissipation of mitochondrial membrane potential, activation of caspase-9 and caspase-3, and decreased exposure to PS. Modulates the distributions of Bax, Bcl-xL, and cytochrome c mediated by H_2_O_2_ in mitochondria and cytochrome. It decreases ROS generation and intracellular Ca^2+^ concentration, caspase-3 signaling and activation, and PS exposure (0.5 or 1 µM). Mechanism: protects platelets from oxidative stress-induced apoptosis by regulating ROS-mediated PI3K/Akt/SK3β signaling [201].
Syringic acid	Phenolic acid	Inhibits clotting factors, decreases the secretion of dense granules, and retraction of the clot. Regulates the downstream signaling pathway of DEP-1/PTP-1B/αIIbβ3/kinases. It decreases the expression of density-enhanced phosphatase-1 (DEP-1)/protein tyrosine phosphatase-1B (PTP1B)/αIIbβ3, as well as the phosphorylation of platelet kinases stimulated with collagen/epinephrine both in vitro and in vivo (5, 10 and 20 µg). It inhibits the secretion of granular components, clot retraction, and FeCl_3_-induced vascular occlusion of the carotid artery. Mechanism: attenuates the development of thrombosis and thromboembolism by inhibiting fibrin, clot formation, clotting factors, and platelet stimulation through DEP-1/PTP1B/αIIbβ3/kinases [202].
Myricerin	Flavonoid	Reduces the ability of platelets to spread on collagen and form thrombi in vitro without affecting hemostasis in vivo. Inhibits the activities of PDI and ERp5 reductase (10, 30, or 100 µM). Mechanism: ERp5 and PDI inhibitor [203].
		Inhibits in vitro PAF-induced platelet adhesion (IC_50_: 13.1 mmol/L) and internal free Ca^2+^ concentration. It inhibits platelet aggregation PAF 1, 2, and 4 nmol/L with IC_50_: 34.8, 85.7, and 118.6 mmol/L. Mechanism: antagonizes the specific binding of the PAF receptor [204].
		Inhibits in vivo 3.6 µg/kg cat blood platelet aggregation. Reduces platelet thrombi in vitro at a concentration of 60 nM. Mechanism: inhibits PGI_2_ synthase [205].
		Inhibits platelet aggregation. Increases the cAMP stimulated by PGI_2_. Inhibits lipoxygenase activity (50 µM). Mechanism: modification of cAMP metabolism through inhibition of phosphodiesterase activity [206].
Genistein	Isoflavonoid	Inhibits in vitro human platelet aggregation, serotonin secretion, and phosphorylation of protein tyrosine induced by collagen and TxA2. It slightly attenuates thrombin-induced protein tyrosine phosphorylation (100 μg/mL). Mechanism: preventive action on TxA2 binding, through inhibition of protein tyrosine phosphorylation [207].
		Inhibits in vitro dose-dependent collagen-induced platelet aggregation, NO production, and TNF-α secretion. Decreased secretion of MCP-1 induced by TNF-α in endothelial cells of the human umbilical vein (50 µM). Mechanism: through TNF-α [208].
		Inhibits platelet aggregation or the release of serotonin-induced by thrombin and Ca^2+.^ Inhibits the COX pathway and PI3 and PI(4,5)P2 (50 µM) production. Mechanism: acts on the passage of inositol phospholipids [209].
		Decreases PAF stimulation of PLC activity at baseline. Inhibits PAF-stimulated platelet aggregation. Inhibits PI3 production and reduces induced phosphorylation (0.5 mM). Mechanism: inhibits tyrosine kinase early in signal transduction by inhibiting PLC. Decreases the activation of PKC and causes a reduction in protein phosphorylation [210].
Glycythein	Isoflavonoid	Inhibits proliferation and synthesis of SMC DNA induced by PDGF-BB (3–10 μmol/L). Mechanism: inhibitory effect on SMC proliferation attenuates said proliferation (basic mechanism involved in atherosclerotic vascular change) [211].
Formononetin	Isoflavonoid	Inhibits ferric chloride-induced arterial thrombus formation in rats and ADP- and thrombin-stimulated platelet aggregation in rats. Prolongs bleeding time and aPTT in mice Mechanism: inhibits platelet aggregation induced by ADP and thrombin and reduces the function of the endogenous clotting pathway [212].
		Inhibits PDGF-BB-induced proliferation and migration of human VSMCs. Inhibits upregulation of cell cycle-related proteins, matrix metalloproteinase, and PDGF-BB-induced phosphorylation of AKT in VSMC (1 µM). Mechanism: suppressive effect on PDGF-BB-stimulated proliferation and migration of VSMC, through inhibition of the AKT signaling pathway [213].
		Increases cell migration, tube formation, and levels of PECAM-1 and VEGF and platelet endothelial cells of the human umbilical vein. Protects against cerebral ischemia and reperfusion injury in rats. Improves cerebrovascular angiogenesis in human umbilical vein endothelial cells (10 and 20 µg/mL). Mechanism: suppresses cell apoptosis and improves cerebrovascular angiogenesis by promoting the expression of VEGF and PECAM-1 [214].
Naringenin	Flavanone	Decreases kidney failure in rats and the lipid profile. Inhibits levels of inflammation markers and pro-oxidants in the kidney of rats. Moderate platelet parameters (50 mg/kg/90 days). Mechanism: improves kidney failure and platelet abnormalities through its antioxidant effects [215].
Hesperetin	Flavanone	Inhibits in vitro platelet aggregation induced with collagen 5 μg/mL and AA 0.5 μmol/L (IC_50_: 20.5 and IC_50_: 69.2). Collagen-induced cytosolic Ca^2+^ mobilization decreases from 10 μg/mL to 20–50 μM. Inhibits collagen-stimulated serotonin secretion at IC_50_: 10.5 and IC_50_: 25.2. Mechanism: inhibition of PLCγ2 phosphorylation and collagen-induced COX-1 activity [216].
		Inhibits in vivo platelet aggregation induced with ADP and collagen (100 mg/kg) [217].
Daidzein	Isoflavonoid	Inhibits in vitro dose-dependent collagen-induced platelet aggregation, NO production, and TNF-α secretion. Decreases secretion of MCP-1 induced by TNF-α in endothelial cells of the human umbilical vein (50 µM). Mechanism: through TNF-α [208].
		Inhibits proliferation and synthesis of SMC DNA induced by PDGF-BB (3–10 μmol/L). Mechanism: inhibits SMC proliferation (basic mechanism involved in atherosclerotic vascular change) [211].
Catechin	Flavonol	Inhibits collagen-induced platelet aggregation and platelet adhesion to collagen (50–100 µmol/L). Mechanism: inhibits platelet function by reducing the production of hydrogen peroxide and PLC [218].
		Inhibits platelet aggregation induced by AA, ADP, and Mepinephrine (200 µg/mL). Decreases MDA production from AA-stimulated platelets (20–200 µg/mL). Mechanism: protects from peroxidative stress [219].
		Ex vivo platelet function improvement in iron-loaded rats, associated with impaired antioxidant defense, including free radical-induced hemolysis (10 mg/kg). Mechanism: through normalization of antioxidant status [220].
Kaempferol-3-glucoside	Flavonol	Inhibits in vitro platelet aggregation induced with 100 μM AA and 10 μg/mL collagen to 22 μM. Inhibits ATP release stimulated with 7 µM ADP and 7 µM epinephrine to 25 µM. Mechanism: inhibits collagen 10 μg/mL induced TxA2 and PG formation L to 5 μM [221].
		Inhibits in vitro platelet aggregation induced by AA 100 μM and collagen 10 μg/mL to 100 μg/mL [222].
		Inhibits in vitro of platelet aggregation induced with AA 150 μM at IC_50_: 24 μM [206].
Luteolin-7 glycoside	Flavone	Inhibits the proliferation of CMLV induced by PDGF-BB 1/2) and DNA synthesis in CMLV (50 uM). Mechanism: inhibits PDGF-BB 1/2 (ERK1/2)-induced extracellular signal-regulated kinase, Akt, and PLC activation [223].
Coumarin	Benzopyrone	Inhibits platelet aggregation and release of ATP from rabbit platelets induced by AA, collagen, ADP, PAF) and U46619 (analogous to TxA2). Inhibits degradation of phosphoinositide caused by collagen and PAF (200 uM). Mechanism: inhibits TxA2 formation and phosphoinositide degradation [224].
		Inhibits platelet aggregation (25.75 ± 4.12%). Shows the percentage of binding with GPIIb/IIIa receptor (0.5 and 2 mM) [225].
		Inhibits AA-induced platelet aggregation. It does not interfere with the function of TxA2 synthase, but they were competitive antagonists of TxA2 receptors and inhibited COX-1 (50 µM). Mechanism: TxA2 stimulates its receptors on platelets, promoting platelet aggregation [226].
Starch	Sugar	Reduces availability of the fibrinogen receptor. Mechanism: prevents the platelet from binding to fibrinogen, by blocking the access of ligands to the platelet fibrinogen receptor [227].
Tocopherol	Tocopherol	Inhibits in vitro the aggregation induced by ADP, AA, PMA. Inhibits in vivo PMA-induced stimulation of PKC (400 to 1200 µL/d). Mechanism: inhibits human platelet aggregation through a PKC-dependent mechanism [228].
		Inhibits platelet aggregation induced by ADP and PMA. Increases NO release, ecNOS activation, and platelet protein SOD content. Decreases the activation of PKC (45 mg of α-tocopherol equivalents). Mechanism: increased NO release, ecNOS activation, and SOD protein content in platelets [229].
Omega-3 and 6	Fatty acid	Decreases in vivo reactivity of P2Y12 by 22.2%. Inhibits ADP-induced platelet aggregation. Mechanism: potentiates platelet response to clopidogrel then percutaneous coronary intervention [230].
		Reduces in vivo thrombin formation and oxidative stress and favorably alters the properties of the fibrin clot (1 g/day of PUFA n-3) [231].
		Increases the total surface load of platelets and attenuates platelet activation (1 to 8 g/day) [232].
Delphinidin	Anthocyanin	Inhibits in vitro the secretion of alpha granules: PF4, β-TG, P-selectin, TGF-β1, RANTES, ATP, and serotonin and CD63 induced with thrombin 0.5 U/mL. Inhibits the secretion of dense granules: ATP and serotonin-induced with thrombin 0.5 U/mL (320 mg/day). Mechanism: inhibits the phosphorylation of the MAPK family stimulated by thrombin 0.5 U/mL. Inhibits the activation of PI3K/Akt, phosphorylation of eNOS, and production of cGMP induced by thrombin 0.5 U/mL. Inhibits in vivo the secretion of alpha granules PF4, β-TG, P-selectin, TGF-β1, RANTES stimulated with thrombin 0.5 U/mL (320 mg/day) [233].
		Inhibits platelet aggregation induced with 5 µM ADP, 2 µg/mL collagen, and 100 µM TRAP. Inhibits in vitro the activation and secretion of P-selectin, CD63, CD40L, αllbβ3, and fibrinogen induced with 200 μM ADP, 10 μg/mL collagen, 1 U/mL thrombin, and 250 μM TRAP. Mechanism: inhibits the phosphorylation of MAPK induced by collagen 25 μg/mL. Inhibits in vivo the formation of the thrombus induced by collagen 100 μg/mL, under controlled flow. Inhibits FeCl_3_-induced thrombus formation at 50 μg/mL [234].
Cyanidin-3-O-glucoside	Anthocyanin	Inhibits in vitro the secretion of alpha granules: PF4, β-TG, P-selectin, TGF-β1, RANTES, ATP, and serotonin and CD63 induced with thrombin 0.5 U/mL. Mechanism: inhibits the phosphorylation of the MAPK family induced by thrombin 0.5 U/mL. Inhibits PI3K/Akt activation, eNOS phosphorylation, and 0.5 U/mL thrombin-induced cGMP production [233]. Inhibits in vivo the secretion of alpha granules PF4, β-TG, P-selectin, TGF-β1, RANTES induced with thrombin 0.5 U/mL at 320 mg/day [233].
		Inhibits platelet aggregation induced with 2.5 µg/mL collagen, 0.1 U/mL thrombin, and 100 µM TRAP-6. Mechanism: GPVI collagen receptor pathway. Inhibits collagen 2.5 µg/mL induced phosphorylation of protein tyrosine at 5–50 µM [235]. Inhibits 0.5–50 µM collagen-induced thrombus formation in vivo, under controlled flow. Inhibits FeCl_3_-induced thrombus formation (5–50 µM) [234].
Pelargonidine-3-O-glucoside	Anthocyanin	Prolongs aPTT and PT. Inhibits thrombin and FXa activity and production in human umbilical vein endothelial cells. Inhibits thrombin-catalyzed polymerization of fibrin and platelet aggregation and anticoagulant effect elicited in mice (10 µM) [236].
		Inhibits in vitro the secretion of alpha granules: PF4, β-TG, P-selectin, TGF-β1, RANTES, ATP, and serotonin and CD63 induced with thrombin 0.5 U/mL. Inhibits the secretion of dense granules, ATP, and serotonin with thrombin 0.5 U/mL. Mechanism: inhibition of the phosphorylation of the MAPK family induced by thrombin 0.5 U/mL. Inhibits PI3K/Akt activation, eNOS phosphorylation, and 0.5 U/mL thrombin-induced cGMP production [233]. Inhibits in vivo the secretion of alpha granules PF4, β-TG, P-selectin, TGF-β1, RANTES induced with thrombin 0.5 U/mL (320 mg/day) [233].

Abbreviations: AA: arachidonic acid, ADP: adenosine diphosphate, Akt: protein kinase B, aPTT: activated partial thromboplastin time, ATP: adenosine triphosphate, cAMP: cyclic adenosine monophosphate, CCL5: type 5 receptor chemokine, CD63: gene encoded by the symbol CD63, VSMC: vascular smooth muscle cells, COX-1: cyclooxygenase 1, DOX: doxorubicin, ecNOS: constitutive endothelial nitric oxide synthase, ERp5 reductase: endoplasmic reticulum protein 5, PLA: phospholipases A2, VWF: factor de von Willebrand, FXa: tissue factor, cGMP: cyclic guanosine monophosphate, GPIb: glycoprotein, IL-1β: interleukin-1-beta, IP3: inositol triphosphate, LDH: lactate dehydrogenase, MAPK: mitogen-activated protein kinase, MCP-1: monocyte chemoattractant protein 1, MDA: malondialdehyde, NO: nitrogen oxide, PAF: platelet activating factor, PDE: phosphodiesterase, PDGF: platelet growth factor, PDI: protein disulfide isomerase, PECAM-1: molecule platelet-endothelium adhesion cells, PGI2: prostacyclin, PI(4,5)P2: phosphatidylinositol-4,5-bisphosphate, PKA: protein kinase A, PKC: protein kinase C, PLC: phospholipase, PMA: phorbol myristate acetate, PS: phosphatidylserine, PT: prothrombin time, RANTES: subfamily chemokine C, ROS: reactive oxygen species, sCD40L: differentiation groups or differentiation antigens, SMC: smooth muscular system, SOD: superoxide dismutase, TGF-β1: transforming growth factor beta-1, TNF-α: tumor necrosis factor alpha, TRAP: thrombin receptor activator for peptide 6, TxA2: thromboxane, VASP: vasodilator-activated phosphoprotein, VEGF: vascular endothelial growth factor, β-TG: ß-thromboglobulin.

## Data Availability

Not applicable.
